# Type I Interferons in SARS-CoV-2 Cutaneous Infection: Is There a Role in Antiviral Defense?

**DOI:** 10.3390/ijms26136049

**Published:** 2025-06-24

**Authors:** Tatiana Mina Yendo, Raquel Leão Orfali, Naiura Vieira Pereira, Natalli Zanete Pereira, Yasmim Álefe Leuzzi Ramos, Joyce Tiyeko Kawakami, Amaro Nunes Duarte-Neto, Mirian Nacagami Sotto, Luiz Fernando Ferraz Silva, Alberto José da Silva Duarte, Maria Notomi Sato, Valeria Aoki

**Affiliations:** 1 Department of Dermatology, Faculdade de Medicina FMUSP, Universidade de Sao Paulo, Sao Paulo 01246-903, SP, Brazil; tatiana.yendo@hc.fm.usp.br (T.M.Y.); naiurav@gmail.com (N.V.P.); natalli@usp.br (N.Z.P.); yasmimleuzzi@gmail.com (Y.Á.L.R.); marisato@usp.br (M.N.S.); valeria.aoki@hc.fm.usp.br (V.A.); 2 Laboratory of Heart Pathology, Heart Institute (InCor), Faculdade de Medicina FMUSP, Universidade de Sao Paulo, Sao Paulo 05403-900, SP, Brazil; joyce.kawakami@incor.usp.br; 3 Department of Pathology, Faculdade de Medicina FMUSP, Universidade de Sao Paulo, Sao Paulo 01246-903, SP, Brazil; amaro.ndneto@hc.fm.usp.br (A.N.D.-N.); mnsotto@usp.br (M.N.S.); burns@usp.br (L.F.F.S.); aduar-te@hcor.com.br (A.J.d.S.D.)

**Keywords:** COVID-19, SARS-CoV-2, skin, STING

## Abstract

SARS-CoV-2, a β-coronavirus, primarily affects the lungs, with non-specific lesions and no cytopathic viral effect in the skin. Cutaneous antiviral mechanisms include activation of TLR/IRF pathways and production of type I IFN. We evaluated the antiviral mechanisms involved in the skin of COVID-19 patients, including skin samples from 35 deceased patients who had contracted COVID-19 before the launch of the vaccine. Detection of SARS-CoV-2 in the skin was performed using transmission electron microscopy and RT-qPCR. Microscopic and molecular effects of the virus in skin were evaluated by histopathology, RT-qPCR, and immunohistochemistry (IHC). The results revealed the presence of SARS-CoV-2 and microscopic changes, including microvascular hyaline thrombi, perivascular dermatitis, and eccrine gland necrosis. There was increased transcription of TBK1 and a reduction in transcription of TNFα by RT-qPCR in the COVID-19 group. IHC revealed reduced expression of ACE2, TLR7, and IL-6, and elevated expression of IFN-β by epidermal cells. In the dermis, there was decreased expression of STING, IFN-β, and TNF-α and increased expression of IL-6 in sweat glands. Our results highlight the role of type I IFN in the skin of COVID-19 patients, which may modulate the cutaneous response to SARS-CoV-2.

## 1. Introduction

Severe acute respiratory syndrome coronavirus-2 (SARS-CoV-2) is a highly transmissible coronavirus responsible for the coronavirus disease 19 (COVID-19) pandemic that began in 2019 in the city of Wuhan, China [1]. It is a single-stranded, sense, 29.9 kilobase, enveloped ribonucleic acid (RNA) virus (envelope derived from the membrane of the endoplasmic reticulum and Golgi apparatus, formed by the budding of the virus from these organelles), containing a helical nucleocapsid [2]. It has six functional genes that encode the replicase, the S protein (spike), the viral envelope, the membrane and the nucleocapsid [1]. Entry into human cell depends on the binding of the viral S protein to the angiotensin-converting enzyme 2 (ACE2) and the activity of serine-proteases such as transmembrane protease serine 2 (TMPRSS2) [3]. Once inside the host cell, the virus uses the cell machinery to replicate itself and then disseminates to other cells. The SARS-CoV-2 virus was detected in different organs, such as lungs, heart, and kidneys, and in the gastrointestinal tract [4].

COVID-19 infection in the skin presents as non-specific manifestations (e.g., pernio-like lesions, urticaria, exanthemas, livedo, vasculitis, among others), associated with immunological and thromboembolic reactions related to the systemic viral infection and the inflammatory reaction to it [5,6,7]. Mild skin lesions were reported by Avancini et al. [8] and Méndez Maestro et al. [9] with spontaneous resolution, and no need for specific treatment. Such mild cutaneous presentation in the context of an established COVID-19 infection involving other organs may be due to inefficient invasion of skin cells or to successful antiviral mechanisms; however, the exact reason remains to be determined.

The antiviral cutaneous immunity involves immunological mechanisms as the skin represents the interface between the environment and the human organism [10]. Toll-like receptors (TLRs) recognize viral DNA, RNA, and proteins and trigger an intracellular signaling pathway that leads to activation of interferon regulatory factors (IRFs) and production of type I interferon (IFN) [11,12]. Cytosolic DNA, originated from DNA damage, mitochondrial stress, and cell death due to viral infection and replication, is recognized by cyclic GMP-AMP synthase (cGAS), that activates stimulator of IFN genes (STING) and activates the TANK-binding kinase 1 (TBK1)-associated pathway, also resulting in the transcription of type I IFN [12,13]. The activation of JAK-STAT pathways by type I IFNs will promote the inhibition of viral protein synthesis and result in the degradation of viral RNA [12,13].

The aim of this study is to explore the role of the antiviral factors and inflammatory components in the skin of a unique cohort composed of severe patients who died from COVID-19 before the vaccine launch, without vaccine-induced immunity against SARS-CoV-2 virus. A better understanding of these mechanisms should provide new insights to elucidate host antiviral defense processes in cutaneous tissue during COVID-19 infection.

## 2. Results

### 2.1. Demographic Data

It was estimated that the introduction of the first SARS-CoV-2 clades in Brazil occurred between 22 February and 11 March 2020, from Europe [14]. A phylogenetic analysis from Brazilian genomes revealed that the main initial clades during the early pandemic phase were clade 1 (nucleotide substitution in spike protein, lineage B.1.1, mainly in Sao Paulo), clade 2 (two nucleotide substitutions in ORF6 and nucleoprotein, lineage B.1.1.33, 16 states in Brazil) and clade 3 (lineage B.1.1.33, mainly in Ceara) in Brazil [14,15]. Our unique cohort included 35 unvaccinated against SARS-CoV-2 patients who died of severe COVID-19 during the early pandemic period, from March to July 2020 in Sao Paulo, Brazil. The healthy control (HC) group comprised the skin of 13 healthy donors (aged between 28–74 years; mean age: 44.84 ± 15.69; 3 males and 10 females).

Demographic data of COVID-19 patients are summarized in Table S1. Briefly, evaluating the body mass index (BMI), our results showed that most patients were of normal weight (*n* = 15/35; 42.8%) or overweight (*n* = 12/35; 34.3%). The main comorbidities in the COVID-19 group were systemic arterial hypertension (*n* = 14/35; 40%), diabetes mellitus (*n* = 8/35; 22.9%), smoking (*n* = 7/35; 20%), and neoplasia (*n* = 6/35; 17.1%), including three cases of breast neoplasia, one case of multiple myeloma, one case of chronic myeloid leukemia, and one case of unspecified leukemia. Other comorbidities included heart disease (*n* = 6/35; 17.1%), vascular disease (deep vein thrombosis, stroke) (*n* = 5/35; 14.3%), asthma (*n* = 3/35; 8.6%), lung disease chronic obstructive disease (*n* = 3/35; 8.6%), kidney and kidney and pancreas transplants (*n* = 2/35; 5.7%), chronic kidney disease (*n* = 2/35; 5.7%), and HIV infection (*n* = 1/35, 2.9%). One patient was pregnant, and two were in the postpartum period (Figure S1).

Laboratory findings of the COVID-19 group: leukocytosis in 45.7% of cases (*n* = 16/34), with values ranging from 4700 to 42,280 leukocytes/mm^3^ (normal range (NR): 4000 to 11,000 leukocytes/mm^3^), and all patients (*n* = 34) had lymphopenia, with a range between 40 and 1050 lymphocytes/mm^3^ (NR: 1500 to 3500 lymphocytes/mm^3^); platelet counts ranged between 4000 and 287,000/mm^3^, with half of the patients presenting thrombocytopenia (*n* = 17/34; 50%) (NR: 150,000 to 400,000/mm^3^); all patients had a D-dimer test (*n* = 31; 1023 to 126,078 ng/mL) above the reference value (NR: <500 ng/mL); R (NR: 0.8 to 1.17) and INR (NR: 0.95 to 1.2) values ranged from 0.92 to 6.73 and 0.99 to 3.08, respectively; 66.7% of patients showed increased fibrinogen (*n* = 14/21; 222 to 8940 mg/dL) (NR: 200 to 393 mg/dL) and only 18.5% of cases showed augmented troponin (*n* = 5/27; 0.005 to 99) (NR: <0.014 ng/mL); four cases did not present increased creatinine (*n* = 4/33; 0.92 to 9.86) (NR: <1.1 mg/dL) and all patients presented increase in C-reactive protein (CRP) (*n* = 30; 23.6 to 464) (normal range: <5 mg/L) (Table S2).

### 2.2. Histological Analysis of Skin (COVID-19 Group)

**Figure 1:**
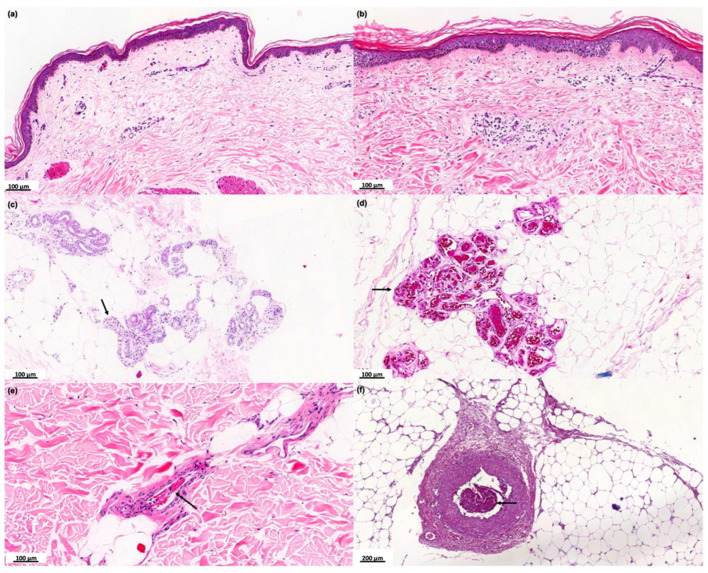
Histopathological analysis of the skin of the COVID-19 group. Photographs of skin specimens from healthy controls (HCs) and COVID-19 patients by hematoxylin and eosin staining. (**a**) Absence of significant histological changes (×100). (**b**) Mild to moderate perivascular dermatitis (×100). (**c**) Vacuolar degeneration of sweat glands cells (black arrows) (×100). (**d**) Necrosis of eccrine glands associated with hyaline thrombi (black arrows) in the peri glandular vessels (×100). (**e**) Hyaline thrombus in dermal vessel (black arrow) (×100). (**f**) Hyaline thrombus in a larger vessel in the hypodermis (black arrow) (×200).

Six of 32 COVID-19 patients did not show any relevant histological changes (Figure 1a). Seven of the 32 cases presented mild to moderate perivascular dermatitis (Figure 1b), 1/32 showed mild perifolliculitis, and 4/32 interstitial hemorrhage; 2/32 cases evidenced ductal ectasia and vacuolar changes in sweat glands (Figure 1c). In 12/32 cases, hyaline thrombi were observed inside the dermis dermal (Figure 1d,e) or hypodermis (Figure 1f) vessels, with or without the presence of the alterations described above.

### 2.3. Transmission Electron Microscopy (COVID-19 Group)

**Figure 2:**
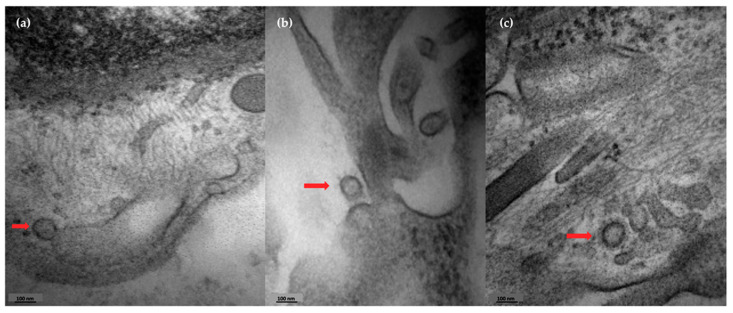
Skin detection of coronavirus by transmission electron microscopy. Photographs of the presence of coronavirus in skin sections from a COVID-19 patient. (**a**) Presence of viral budding (red arrow). (**b**,**c**) Viral particles detaching from cellular structures (red arrows).

Skin samples from four COVID-19 subjects with positive viral load were analyzed by transmission electron microscopy to evaluate the presence of viral elements in ultrastructural analysis. We observed the presence of viral buddings (Figure 2a) and the complete detachment of the virus from cellular cisterns, with the presence of external elements that form the crown of the coronavirus (Figure 2b,c).

### 2.4. Evaluation of the Expression of Viral Load, Viral Receptor Genes, Antiviral, and Pro-Inflammatory Markers in the Skin of COVID-19 Group by RT-qPCR

**Figure 3:**
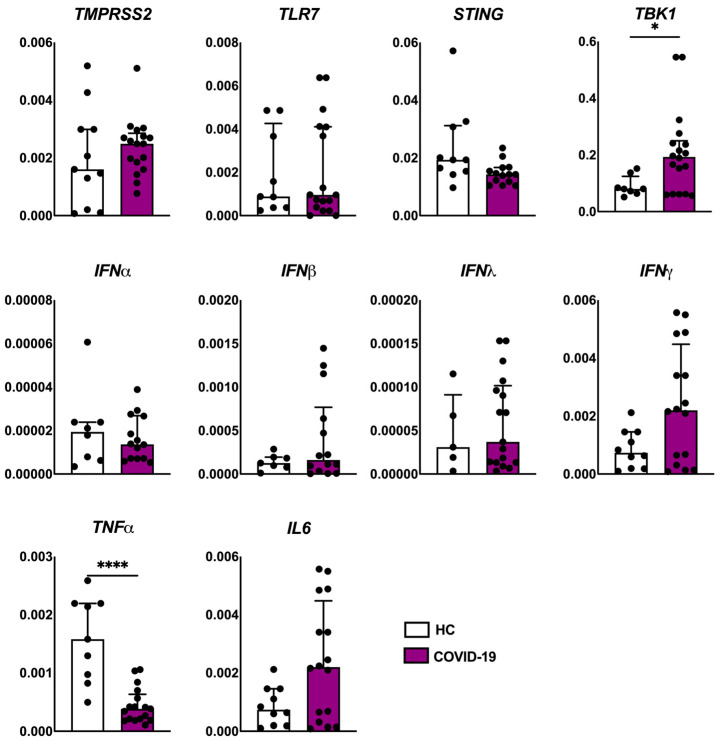
Evaluation of the expression of viral receptor (*TMPRSS2* and *TLR7*), antiviral markers (*STING*, *TBK1*, *IFNα*, *IFNβ*, *IFNλ* and *IFNγ*), and pro-inflammatory markers (*TNFα* and *IL6*) genes in the skin of patients with COVID-19 (*n* = 18) compared to healthy controls (HCs, *n* = 10). Values are expressed as median with interquartile range. * *p* < 0.05; **** *p* < 0.0001.

The viral load detection was confirmed in 4/15 patients of the COVID-19 group. The analysis of gene expression associated with viral recognition by host cells, antiviral and pro-inflammatory markers revealed that the COVID-19 group showed an increased expression of *TBK1* transcripts compared to the healthy control group. We also observed a decreased expression of the tumor necrosis factor α (*TNFα*) gene (Figure 3). We detected a positive correlation between *IFNβ* and *IFNγ* (*p* < 0.02), *IFNβ* and *IFNα* (*p* < 0.004), *IFNβ* and *IL6* (*p* < 0.02)—Figure S3.

### 2.5. Analysis of Viral Receptors, Antiviral Proteins, and Pro-Inflammatory Cytokines Expressed in COVID-19 Skin Cells by Immunohistochemistry

**Figure 4:**
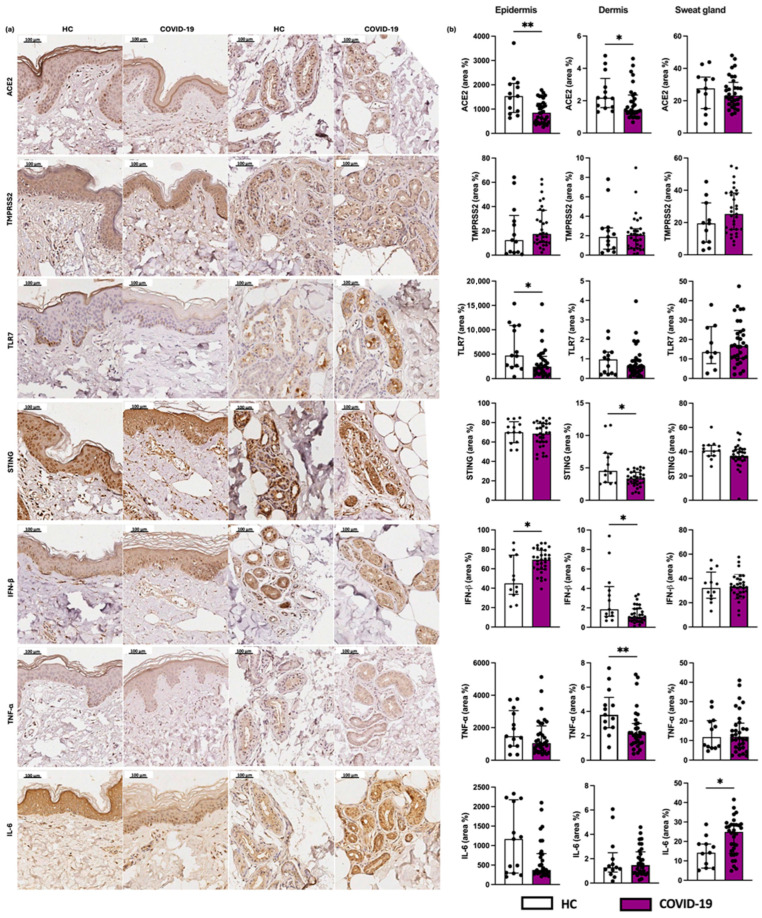
Evaluation of the expression of viral receptors. (**a**) Photographs of skin specimens from healthy controls (HCs) and COVID-19 patients: ACE2, TMPRSS2, and TLR7 (×200). (**b**) Expression of ACE2, TMPRSS2, and TLR7 in the epidermis (area %), dermis (area %), and sweat glands (area %) in COVID-19 patients (*n* = 32) in comparison to the HC group (*n* = 13). Values are expressed in median with interquartile range. * *p* < 0.05; ** *p* < 0.01.

We observed decreased expression of the viral receptors TLR7 and ACE2 in the epidermis of COVID-19 patients compared to HCs (Figure 4). When evaluating the antiviral markers, we found a reduced expression of STING and IFN-β at dermal level of the COVID-19 group, and an increased expression of IFN-β in the epidermis of the COVID-19 group compared to the HC (Figure 4).

Regarding pro-inflammatory cytokines, we found a reduced expression of TNF-α in the dermis of the COVID-19 group compared to HC. We also observed an increased IL-6 expression by the sweat gland cells in patients of the COVID-19 group (Figure 4).

## 3. Discussion

SARS-CoV-2 infection in humans is characterized by a massive, multisystemic cytokine storm [16]. Interestingly, the skin involvement shows mild to moderate clinical manifestations, even in the presence of severe multiorgan compromise. The purpose of the current study was to evaluate the skin of post-mortem samples from patients with severe COVID and extensive multiorgan involvement. In this study, we revealed the presence of SARS-CoV-2 and microscopic changes (microvascular hyaline thrombi, perivascular dermatitis, and eccrine gland necrosis) in the skin of severely infected patients. Of note, we reported elevated transcription of TBK1 and a reduction in transcription of *TNFα* by RT-qPCR, and also a reduced expression of ACE2 and TLR7, and elevated expression of IFN-β by epidermal cells utilizing IHC. Moreover, there was dermal decreased expression of STING, IFN-β, and TNF-α and increased expression of IL-6 in sweat glands.

In our case series, the profile of COVID-19 patients showed a predominance of women (19/35), aged between 31 and 80 years (30/35), and not obese (28/35). All patients died due to COVID-19 complications between March and July 2020 and did not present any vaccine-induced immunity against SARS-CoV-2. The first case of COVID-19 in Brazil was confirmed in March 2020, initiating the first COVID-19 wave in Brazil. The first Brazilian peak of COVID-19 cases was caused by the ancestral SARS-CoV-2 (lineage B.1), which preceded the variants of concern. A total of 3,817,247 were confirmed in Brazil with 5.06% of hospitalization rate during the first wave [17]. Souza et al. [17] reported that the main risk factors for a significant higher lethality rate between 26 February to 10 August 2020 in Brazil were older age and invasive mechanical ventilation. Other previous publications showed that most patients who deceased from COVID-19 were male and elderly, with obesity as a risk factor for a fatal outcome [18]. Such differences may be due to limited sample sizes, obtained during the most dramatic initial period of the pandemic, before COVID-19 vaccination.

We reported at least one comorbidity (especially systemic arterial hypertension and diabetes mellitus) in 24/35 patients. These two aforementioned diseases increase the risk of death from COVID-19 infection through mechanisms that augment viral replication and infection and induce the production of proinflammatory cytokines, resulting in a cytokine storm and a prothrombotic state [16]. Laboratory alterations revealed elevated D-dimer, R, INR, and a prothrombotic state; 12 of the 32 cases showed histopathological alterations compatible with thrombi in vessels of the dermis and hypodermis. Thrombi in dermal and hypodermal vessels were also found in cases of livedo racemosa, which was observed in some COVID-19 severe cases, associated with coagulopathy [19].

Our results showed a lower expression of ACE2 by the epidermal cells of the COVID-19 group, when compared to HCs. Lower availability of this receptor on the cell membrane due to its prior binding to SARS-CoV-2 and internalization of the virus, or negative feedback generated by the presence of the virus in the skin, may represent an attempted antiviral response. The entry of SARS-CoV-2 into human cells occurs due to its binding to ACE2 and the activity of serine proteases such as TMPRSS2 [3,20,21,22]. These proteins are present in skin cells, with no statistical difference in TMPRSS2 expression between the COVID-19 and control groups. TMPRSS2 cleaves the viral spike protein (S) into S1 and S2 subunits. The S1 protein binds to ACE2 and S2 promotes the fusion of viral and cellular membranes, allowing the internalization of the virus [3,20,21,22].

Our striking finding was the detection of SARS-CoV-2 in clinically unaffected skin from the COVID-19 group, as demonstrated by transmission electron microscopy and RT-qPCR. Previous reports showed the presence of the virus in skin with clinical alterations after the identification of the SARS-CoV-2 S protein by immunohistochemistry in the cytoplasm of dermal vessels, secretory and excretory eccrine cells in acral perniosis-like lesions [23,24], and also in dermal vessels of skin biopsies without clinical alterations [25]. Our findings support the hypothesis that despite SARS-CoV-2 cutaneous infection, the epidermal response may prevent viral replication and intense local inflammation. The presence of SARS-CoV-2 in the skin did not reflect a direct cytopathic effect on cutaneous cells, nor trigger an inflammatory response, once there was absence of severe clinical or histopathological alterations in our samples.

As for the inflammatory and thrombotic histopathologic findings in our COVID skin samples, we hypothesize that the virus itself, or circulating inflammatory cells may release proinflammatory and prothrombotic mediators that trigger these responses in the skin of COVID patients. Previous reports of COVID patients with cutaneous manifestations also demonstrated similar findings [6,19].

**Figure 5:**
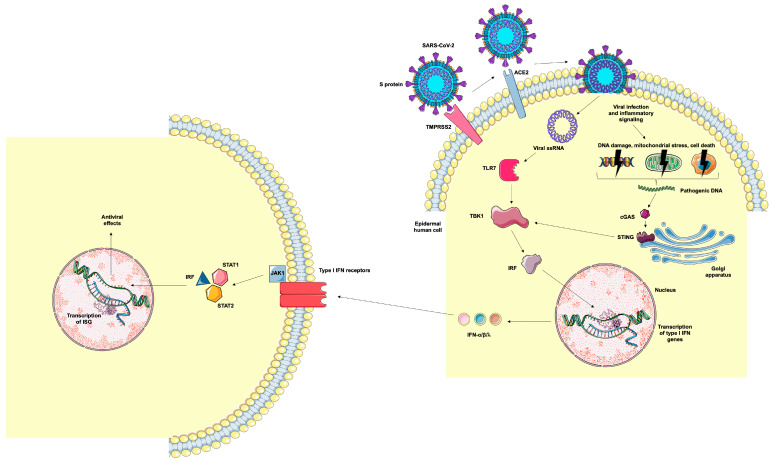
Representation of epidermal human cell infection by SARS-CoV-2 and antiviral mechanisms. TMPRSS2 cleaves the viral S protein into S1 and S2 subunits. The S1 protein binds to ACE2 and S2 promotes the fusion of viral and cellular membranes, allowing the internalization of the virus. Viral RNA binds to TLR7, resulting in the activation of TBK1 and IRF, which are translocated to the cell nucleus, resulting in the gene transcription of type I IFN (α, β, and λ). Cellular infection by the virus and the inflammatory milieu can lead to DNA damage, mitochondrial stress, and cell death, resulting in the generation of cytosolic DNA. These pathogenic DNAs bind to cGAS, which activates the STING signaling pathway, resulting in the transcription of type I IFN genes. Type I IFNs bind to cellular receptors, activating the JAK-STAT intracellular pathway that results in the translocation of IRF to the nucleus and the transcription of ISGs. Image provided by Servier Medical Art (https://smart.servier.com/), accessed on 20 May 2025, licensed under CC BY 4.0 (https://creativecommons.org/licenses/by/4.0/), accessed on 20 May 2025.

Our results reinforce the hypothesis that the skin may find antiviral mechanisms preventing virus replication and survival. In COVID patients, we observed an increased transcription of *TBK1*, associated with elevated expression of IFN-β in the epidermis, despite low expression of markers involved in the mechanisms of antiviral response in the dermis. Additionally, we also detected a lower expression of TLR7 in the skin, corroborating the relevance of the epidermal STING signaling pathway, preventing the entrance of the virus through the skin barrier. There are reports of protective mechanisms against infections due to its close relationship with the external environment [10]. The initial cutaneous antiviral mechanisms are associated with the activation of the innate immune response, from the binding of viral RNA to cellular receptors such as TLR7, resulting in the activation of TBK1 and IRF, which are translocated to the cell nucleus, resulting in the gene transcription of type I IFN (α, β, and λ) [11]. The mitochondrial stress and DNA damage caused by the virus generates pathogenic cytosolic DNA. It binds to cGAS in the cytosol and can also activate STING, which is located in the Golgi apparatus, and consequently TBK1 signaling pathway, resulting in the transcription of type I IFN genes [13]. Type I IFN is an important cytokine involved in the antiviral innate and cellular immunity and it is produced by plasmacytoid dendritic cells and leukocytes. Type I IFNs bind to cellular receptors that activates Janus kinase (JAK), which phosphorylate signal transducers and activators of transcription (STATs) and interferon regulatory factors (IRFs), that trigger the transcription of ISGs [26]. This mechanism will induce innate inflammatory responses and it acts as a bridge to activate of adaptative immune system, that will inhibit viral protein synthesis and will lead to the degradation of viral RNA [11,27,28]. Impaired type I IFN responses, associated to an exacerbated inflammation, are connected to life-threatening COVID-19 [27,29,30]. Domizio et al. [31] demonstrated that cGAS takes an important part in the aberrant type I IFN response against SARS-CoV-2. The presence of phosphorylated STING and high levels of ISGs and pro-inflammatory cytokines such as IL-1, IL-6, and TNF- α, especially in IFN-β-producing perivascular macrophages and damaged endothelial cells, were demonstrated in skin lesions of patients with moderate to severe COVID-19. However, they did not present any data related to the epidermis. Moreover, acquired neutralizing auto-antibodies against type I IFN (α and ω) were identified in at least 10% of severe cases of COVID-19 [32]. This adaptative autoimmunity leads to an impaired innate antiviral immunity, resulting in severe pneumonia due to COVID-19 and a delayed clearance of the virus in cases with high levels of anti-type I IFN [33,34]. These facts indicate that type I IFNs are important for the protection against SARS-CoV-2 and the elevated levels of IFN- β in the epidermis may have a role in protecting the skin against this virus, reducing its replication, transmission, and cell damage. However, in other organs of severe COVID-19 patients, SARS-CoV-2 infection results in an aberrant type I IFN response as a result of viral evasion mechanisms [27]. Non-structural SARS-CoV-2 proteins interact with antiviral-associated proteins, such as proteins associated to the pathway that results in the production of type I IFN and ISGs, resulting in an inefficient immune response against the virus [27,35,36,37,38]. For instance, in the lungs, the first and main organ affected by this virus, different viral proteins induce and imbalance between type I IFN and pro-inflammatory cytokines, resulting in cell damage and amplification of the inflammation [39]. There may be unique mechanisms in the epidermis that prevent the imbalance between type I IFN and pro-inflammatory cytokines, resulting in an efficient protection against SARS-CoV-2. Uncovering these mechanisms may be a key for the development of new antiviral therapeutics (Figure 5).

We also reported decreased expression of STING, IFN-β, and TNF-α in the dermis of COVID patients without clinical cutaneous lesions, mostly in endothelial and inflammatory cells. Microscopic dermal vascular alterations were also observed in our cases. Vascular injury in the context of COVID-19 has been demonstrated in numerous organs, including the skin [25,40,41]. The production of IFN-β by perivascular macrophages and damaged endothelial cells in skin lesions of COVID-19 patients were demonstrated, alongside with elevated levels of IL-1, IL-6, and TNF-α, resulting in a disrupted type I IFN and pro-inflammatory cytokine response [31]. In non-cutaneous lesions cases, pro-inflammatory cytokines present in the circulation of severe patients may have downregulated the STING pathway or did not have enough time to evolve, resulting in a diminished expression of type I IFN, as previously demonstrated by Blanco-Melo et al. [29]. We believe that the epidermis and dermis present individual behaviors when facing SARS-CoV-2. The dermis is mainly composed of fibroblasts, extracellular matrix, nerves, and small vessels. It is directly influenced by serum elements, such as circulating pro-inflammatory proteins during the cytokine storm, and the translocation of immune cells from the circulation to the skin initiates in the dermis. These elements interfere in the dermis microenvironment and may influence STING signaling, as well as type I IFN production and its cellular response. The sweat glands are also located in the dermis and we observed a peri-eccrine inflammatory infiltrate with elevated IL-6 expression by these structures. The epidermis is mainly composed of keratinocytes, is separated from the dermis by the basement membrane zone, and does not contain vessels. This creates a different environment in the epidermis, with a reduced influence of systemic inflammation. This compartmentalization of the epidermis may favor the production of IFN-β, promoting an effective antiviral response that is sufficient to control the virus replication in the skin and avoid cutaneous cells damage, thus resulting in a transmission blockage through this organ.

We demonstrated a reduction in both *TNF-α* transcripts in the skin, as well as in the expression of TNF-α by dermal cells, a decreased expression of IL-6 in epidermal cells, and an augmented expression of this interleukin in sweat glands. These findings may be due to the activation of the antiviral immune response pathway in skin cells, thus reflecting the non-participation of this organ in feeding the systemic inflammatory state. However, this pathway could not prevent the skin damage secondary to systemic inflammatory and prothrombotic activity of the cytokine storm. It is known that in severe COVID-19 cases, cytokines such as TNF-α and IL-6 are released in great amounts and associated with an intense and uncontrolled inflammation called the cytokine storm [16]. This phenomenon corresponds to a hyperinflammatory state that may result in multiple organ failure and fatal outcomes [1,24].

As limitations of our study, at pandemic time there were restrictions on access to our hospital areas to avoid a broader spread of this disease. We also faced difficulties in acquiring individual protection equipment and research inputs. Only critically severe COVID-19 patients were admitted to our hospital, so we did not have access to patients with mild COVID-19 to be included in the present study. Also, due to difficulties with individual security, we chose to perform TEM analysis only in the 4/15 patients with positive viral load detected by RT-qPCR.

In conclusion, our study evidenced that the skin may serve as a model for studying antiviral mechanisms against the SARS-CoV-2 virus, offering new perspectives in therapies for COVID-19 infection, exploiting the production of type I IFN, as a target to be explored.

## 4. Materials and Methods

### 4.1. Sample Collection

All patients were admitted to the Hospital das Clinicas HCFMUSP, Faculdade de Medicina, Universidade de Sao Paulo, Sao Paulo, SP, BR, and submitted to nasopharyngeal, oropharyngeal, or tracheal swabs upon hospital admittance. When admitted in severe conditions just before death, the swab was collected immediately after death. Cases with negative reverse transcriptase polymerase chain reaction (RT-PCR) in upper airway secretion were also submitted to post-mortem confirmation by lung RT-PCR for SARS-CoV-2 using primers and probes set for envelope and nucleoprotein genes. All clinical information during hospitalization was collected retrospectively from the patient’s medical records.

The minimally invasive autopsy was performed at the Image Platform at Autopsy Room Research Facility—Sao Paulo Autopsy Service and Faculdade de Medicina FMUSP, Universidade de Sao Paulo, Sao Paulo, SP, BR. A total of 35 patients were enrolled for minimally invasive autopsies between March and July 2020, as described elsewhere [42]. Skin samples were collected using a 4 mm punch biopsy from the left thigh. None of our patients had specific skin lesions related to COVID-19 infection. Exclusion criteria included patients without positive investigation for SARS-CoV-2, patients with other viral infectious diseases, and patients whose families did not consent to the minimally invasive autopsy. Skin samples of 13 healthy volunteers, collected before the pandemic, from the Laboratory of Medical Investigation in Dermatology and Immunodeficiencies (LIM-56) biorepository, Department of Dermatology, Faculdade de Medicina FMUSP, Universidade de Sao Paulo, Sao Paulo, SP, BR. This study was approved by the institutional and federal ethics boards, and informed consent was obtained from a family member of each subject involved in the study. Demographic information is shown in Table S1.

### 4.2. Histological Analysis

Skin samples were fixed in buffered 10% formalin, embedded in paraffin, and 4 μm sections were stained with hematoxylin and eosin. The samples were analyzed by optical microscopy [43].

### 4.3. Transmission Electronic Microscopy

Ultrastructural changes and the presence of the virus in skin cells were qualitatively analyzed using the transmission electron microscopy technique in four cases of the COVID-19 group. The preparation of the fragments for analysis by transmission electron microscopy was carried out according to the guidelines of Mangini et al. [44]. A camera (Jeol© JEM 1010 EM 80 kV; Tokyo, Japan) attached to the transmission electron microscope (Philips Tecnai 10 80 kV, Hillsboro, OR, USA) was used to document the images.

### 4.4. Expression of mRNA and Viral Load by RT-qPCR

Total RNA was extracted from skin tissues using a RNeasy Plus Mini Kit (Qiagen, Valencia, CA, USA), and reverse transcription was performed with an iSCRIPT Reverse Transcriptase Kit (Bio-Rad, Hercules, CA, USA). Primers used in the RT-PCR assay were: *TMPRSS2* gene: forward primer (5′-AATCGGTGTGTTCGCCTCTAC-3′) and reverse primer (5′-CGTAGTTCTCGTTCCAGTCGT-3′); *TLR7* gene: forward primer (5′-AATGTCACAGCCGTCCCTAC-3′) and reverse primer (5′-GCGCATCAAAA-GCATTTACA-3′); *STING* gene: forward primer (5′- ATATCTGCGGCTGATCCTGC-3′) and reverse primer (5′-GGTCTGCTGGGGCAGTTTAT-3′); *TBK1* gene: forward primer (5′-GCAGTTTGTTTCTCTGTATGGC-3′) and reverse primer (5′-AATGTTACCCCAATGCTCCA-3′); *IFNα* gene: forward primer (5′-AAATACAGCCCTTGTGCCTGG-3′) and reverse primer (5′-GGTGAGCTGGCAT-ACGAATCA-3′); *IFNβ* gene: forward primer (5′-CATTACCTGAAGGCCAAGGA-3′) and reverse primer (5′-CCATTGTCCAGTCCCAGAGG-3′); *IFNλ* gene: forward primer (5′-CGCCTTGGAAGAGTCACTCA-3′) and reverse primer (5′-GAAGCCTCAGGTCCCAATTC-3′); *IFNγ* gene: forward primer (5′-TGTCGCCAGCAGCTAAAACA-3′) and reverse primer (5′-TGCAGGCAG-GACAACCATTA-3′); *TNFα* gene: forward primer (5′-CCCAGGCAGTCAGATCATCTTC-3′) and reverse primer (5′-GCTTGAGGGTTTGCTACAACAT-3′); *IL6* gene: forward primer (5′-CCTGAGAAAGGAGACATGTAA-3′) and reverse primer (5′-GGCAAGTCTCCTCATTGAATCC-3′); *SARS-CoV-2* gene: forward primer (5′-CAGGTACGTTAATAGTTAATAGCGT-3′) and reverse primer (5′-ATATTGCAGCAGTACGCACACA-3′); *GAPDH* gene: forward primer (5′-GAAGGTGAAGGTCGGAGT-3′) and reverse primer (5′-GAAGATGGTGATGG-GATTTC-3′) and probe forward sequence (FAM-ACACTAGCCATCCTTACTGCGCTTCG).

GAPDH mRNA levels in all samples were used to normalize the mRNA content. PCR was performed in an Applied Biosystems 7500 system using specific primers and SYBR Green (Applied Biosystems, Carlsbad, CA, USA) fluorescence detection reagents. The cycling protocol consisted of 10 min at 95 °C, followed by 40 cycles of 15 s at 95 °C and 60 s at 60 °C. The amplification results were visualized and analyzed using Sequence Detection System (SDS) software version 1. (Applied Biosystems). Normalized expression was calculated as previously described by Livak [45].

### 4.5. Immunohistochemistry for Evaluation of the Expression of Viral Receptors, Anti-Viral Proteins, and Inflammatory Cytokines by Skin Cells

To investigate the expression of ACE2, TMPRSS2, STING (also known as TMEM173), IFN-β, TLR7, IL-6, and TNF-α in specimens from the COVID-19 and the HC groups, the immunohistochemistry technique was used as previously described [20]. The description of the primary antibodies used for the reactions is presented in Table S3. Total tissue distribution of the described antibodies was calculated by dividing the stained area by the total measured area within the epidermis, dermis, or sweat glands. Stained specimens were scanned using a Pannoramic Scan 3DHistech scanner (3DHistech Ltd., Budapest, Hungary). Photographs were analyzed utilizing Image-Pro Plus version 4.5.0.29 (Media Cybernetics Inc., Bethesda, MD, USA) [46,47].

Quantification was carried out with Image-Pro Plus software version 4.5.0.29 (Media Cybernetics) using standardized intensity threshold parameters. For each marker, three representative microscopic fields per sample were analyzed, considering: (i) positive area (µm^2^) corresponding to the area stained with 3,3’-diaminobenzidine above a previously defined intensity threshold, (ii) total tissue area (manually demarcated area), and (iii) Integrated Optical Density (IOD), the average staining intensity. Quantitative analyses were expressed as percentage of immunolabeled area and IOD normalized by total area. All the analyses were carried out using the same acquisition and segmentation parameters between the groups, ensuring reproducibility and comparability of the data.

### 4.6. Statistical Analysis

The Mann–Whitney U-test or Kruskal–Wallis test with Dunn’s post hoc tests were utilized to compare two or three sets of data, respectively. Correlations were established using the Spearman non-parametric correlation test. Differences between groups were considered statistically significant when *p* < 0.05.

## Data Availability

The original contributions presented in this study are included in the article/Supplementary Material. Further inquiries can be directed to the corresponding author.

## Supplementary Materials

The following supporting information can be downloaded at: https://www.mdpi.com/article/10.3390/ijms26136049/s1.

## Abbreviations

The following abbreviations are used in this manuscript:

ACE2	Angiotensin-converting enzyme 2
BMI	Body mass index
cGAS	Cyclic GMP-AMP synthase
CRP	C-reactive protein
COVID-19	Coronavirus disease 19
IFN	Interferon
IHC	Immunohistochemistry
IL	Interleukin
IRF	Interferon regulatory factor
ISG	Interferon-stimulated-gene
RNA	Ribonucleic acid
RT-PCR	Reverse transcriptase polymerase chain reaction
RT-qPCR	Reverse transcriptase quantitative polymerase chain reaction
S protein	Spike protein
SARS-CoV-2	Severe acute respiratory syndrome coronavirus-2
STAT	Signal transducers and activators of transcription
STING	Stimulator of IFN genes
TBK1	TANK-binding kinase 1
TLR	Toll-like receptor
TMPRSS2	Transmembrane protease serine 2
TNF	Tumor necrosis factor
