# Supplementary material for: Type I Interferons in SARS-CoV-2 Cutaneous Infection: Is There a Role in Antiviral Defense?
Source: Int J Mol Sci. 2025 Jun 24;26(13):6049. doi: 10.3390/ijms26136049 (PMC12249743; doi:10.3390/ijms26136049)
Supplement: Supplementary file 1 [file ijms-26-06049-s001.zip › Table S1.pdf]

Table S1: Characteristics of patients in the COVID-19 Group.

Age (years old)	8 – 88 (51.4 ± 18.91)
Gender	19 Female: 16 Male
Ethnicity (n/%)	White (30/85.7%)<br>Mixed race (3/8.6%)<br>Black (2/5.7%)
BMI (n/%)	Underweight (≤18.5 kg/m^2) (1/2.9%)<br>Normal (18.6 – 24.9 kg/m^2) (15/42.9%)<br>Overweight (25 – 29.9 kg/m^2) (12/34.2%)<br>Obesity grade I (30 – 34.9 kg/m^2) (5/14.2%)<br>Obesity grade II (35 – 39.9 kg/m^2) (1/2.9%)<br>Obesity grade III (≥40 kg/m^2) (1/2.9%)
