# Supplementary material for: Type I Interferons in SARS-CoV-2 Cutaneous Infection: Is There a Role in Antiviral Defense?
Source: Int J Mol Sci. 2025 Jun 24;26(13):6049. doi: 10.3390/ijms26136049 (PMC12249743; doi:10.3390/ijms26136049)
Supplement: Supplementary file 1 [file ijms-26-06049-s001.zip › Table S2.pdf]

Table S2: Laboratory results of 35 patients from COVID-19 Group.

Case	Leucocytes (/mm^3)	Lymphocytes (/mm^3)	Platelets (/mm^3)	D-dimer (ng/mL)	R	INR	Fibrinogen (mg/dL)	Troponin T (ng/mL)	Creatinin (mg/dL)	CRP (mg/L)
1	16150	646	139000	NA	NA	NA	NA	NA	NA	23.60
2	10860	1050	176000	28435	1.17	1.30	NA	NA	5.23	NA
3	13940	310	273000	89513	1.15	1.06	538	0.11	3.45	110.49
4	27030	540	223000	NA	0.92	1.34	NA	0.99	5.08	324.8
5	9980	400	126000	2026	NA	1.01	410	0.031	1.04	175.5
6	7240	70	135000	NA	64.10	2.14	403	NA	7.14	351.4
7	14080	560	58000	21384	1.13	1.12	418	0.015	3.54	333.6
8	17880	390	235000	2058	1.78	1.69	NA	0.009	2.12	NA
9	12800	260	96000	84864	4.61	1.24	684	0.03	4.75	295
10	10010	190	222000	1374	2.57	1.05	NA	0.071	6.35	359
11	6460	40	118000	4117	7.90	3.08	NA	NA	6.21	83
12	10760	200	48000	5050	1.16	1.57	376	0.029	1.11	191
13	14530	650	158000	6440	1.12	1.11	434	1.17	8.49	421
14	10250	920	180000	3943	1.11	1.19	NA	0.214	4.09	349
15	15050	290	222000	3093	1.07	1.25	664	0.005	5.25	44
16	12100	350	187000	126078	1.66	1.43	235	0.046	3.55	373
17	8090	80	80000	16308	1.25	2.42	NA	0.525	4.38	NA
18	7900	480	206000	62041	1.15	1.17	312	NA	1.34	45
19	24480	734	145000	54153	1.28	1.75	513	0.342	1.31	267
20	12740	90	50000	120881	2.34	0.99	NA	0.022	4.95	240
21	4700	60	4000	19271	2.42	2.10	645	0.18	7.15	279
22	8480	270	65000	2374	1.91	1.10	491	0.15	4.97	464
23	7790	370	195000	1023	0.94	1.07	480	0.005	0.98	49
24	14650	700	261000	2322	1.09	1.24	894	0.009	3.33	165
25	10160	450	226000	4021	1.21	1.39	NA	0.005	1.00	106
26	6320	60	138000	5867	8.50	1.59	310	0.029	1.16	128
27	NA	NA	NA	NA	NA	NA	NA	NA	NA	NA
28	17120	180	113000	13762	2.10	1.22	349	1.08	9.86	44
29	8880	160	287000	14295	9.50	1.25	389	0.1	4.39	215
30	5520	290	99000	3199	1.57	1.48	480	0.032	2.00	163
31	9710	200	147000	21725	6.73	1.51	NA	NA	2.64	352
32	42280	420	123000	37317	8.40	3.06	222	0.158	2.39	229
33	10980	769	185000	11513	1.06	1.1	NA	NA	1.57	136.5
34	11360	550	285000	7156	0.92	1.06	NA	0.06	3.51	398.6
35	14280	860	158000	48330	0.98	NA	NA	0.047	0.92	NA

NA = not available
