# Supplementary material for: Type I Interferons in SARS-CoV-2 Cutaneous Infection: Is There a Role in Antiviral Defense?
Source: Int J Mol Sci. 2025 Jun 24;26(13):6049. doi: 10.3390/ijms26136049 (PMC12249743; doi:10.3390/ijms26136049)
Supplement: Supplementary file 1 [file ijms-26-06049-s001.zip › Table S3.pdf]

Table S3: Antibody specifications, dilution and detection system utilized in the study.

Antibody	Dilution	Company	Detection system	Positive control
ACE2	1:500	Sigma, HPA000288	Novolink Max Polymerer Detection system, code K0690, Leica Biosystems, Newcastle Upon Tine, UK	COVID-19+ lung
TMPRSS2	1:200	Abcam, ab92605	Reveal Biotin-free Polyvalent DAB, code SPD125, Spring Bioscience, San Francisco, CA, USA	Prostate
TMEM 173 (STING)	1:200	Abcam, ab92605	Novolink Max Polymerer Detection system, code K0690, Leica Biosystems, Newcastle Upon Tine, UK	Breast carcinoma
TLR7	1:100	Abcam, ab124928	Reveal Biotin-free Polyvalent DAB, code SPD125, Spring Bioscience, San Francisco, CA, USA	Amygdala
IFN-β	1:200	Abcam, ab180616	Novolink Max Polymerer Detection system, code K0690, Leica Biosystems, Newcastle Upon Tine, UK	Kaposi's sarcoma
IL-6	1:50	Proteintec, 21865-1-AP	Reveal Biotin-free Polyvalent DAB, code SPD125, Spring Bioscience, San Francisco, CA, USA	Amygdala
TNF-α	1:300	Abcam, ab9635	Novolink Max Polymerer Detection system, code K0690, Leica Biosystems, Newcastle Upon Tine, UK	Amygdala
