# Supplementary figures and images for: Type I Interferons in SARS-CoV-2 Cutaneous Infection: Is There a Role in Antiviral Defense?
Source: Int J Mol Sci. 2025 Jun 24;26(13):6049. doi: 10.3390/ijms26136049 (PMC12249743; doi:10.3390/ijms26136049)

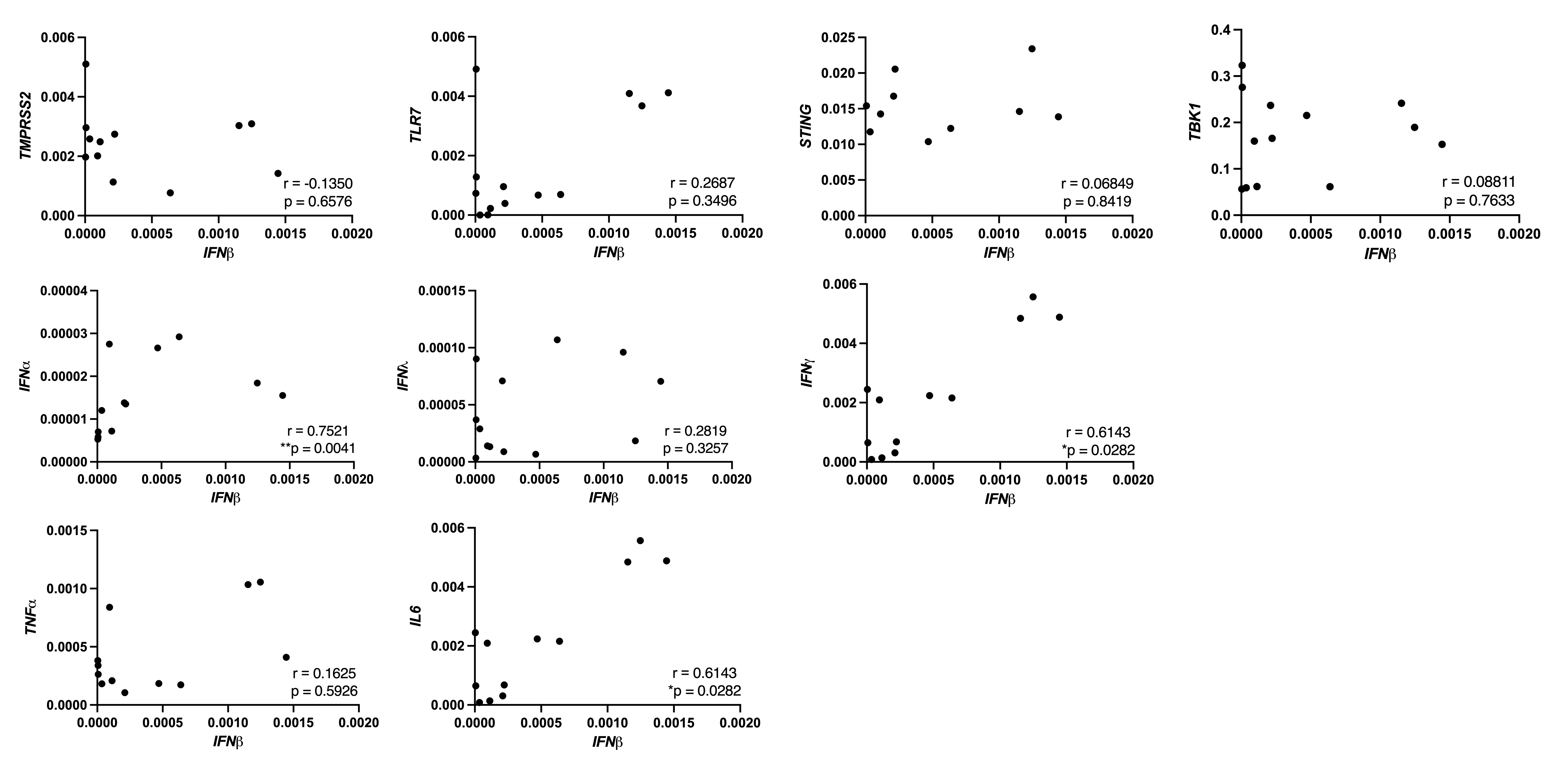

Supplement: Supplementary file 1 [file ijms-26-06049-s001.zip › Figure S3 R1 2025.tiff]
